# The Intramolecular Diels-Alder Reaction of Diarylheptanoids — Quantum Chemical Calculation of Structural Features Favoring the Formation of Phenylphenalenones

**DOI:** 10.3390/molecules19045231

**Published:** 2014-04-23

**Authors:** Yulia Monakhova, Bernd Schneider

**Affiliations:** 1 Department of Chemistry, Saratov State University, Astrakhanskaya Street 83, Saratov 410012, Russia; E-Mail: yul-monakhova@mail.ru; 2 Max Planck Institute for Chemical Ecology, Hans Knöll-Str. 8, Jena 07745, Germany

**Keywords:** diarylheptanoids, (4+2)-cycloaddition, Diels-Alder reaction, phenylphenalenones, quantum chemistry, 6-31G*, molecular orbitals

## Abstract

Diarylheptanoids have been reported as biosynthetic precursors of phenylphenalenones in plants. Quantum chemical calculations of molecular geometry and orbitals were used to elaborate which structural features are required to determine if diarylheptanoids can undergo an intramolecular Diel-Alder reaction to form phenylphenalenones. The computational data showed that an ortho-quinone- or a hydoxyketone-bearing ring A, containing the dienophile moiety, and a heptadiene chain with conjugated cisoid double bonds at C-4/C-6 and a saturated segment consisting of two sp^3^-carbon atoms, are required. Only four diarylheptanoids out of eighteen studied compounds proved to be suitable candidates. Among them are two 3,5-dideoxy compounds and two other compounds oxygenated only at C-3, suggesting that lachnanthocarpone, a representative of the 6-oxygenated phenylphenalenones, and anigorufone, a representative of the 6-deoxy phenylphenalenones, are not connected via a precursor-product relationship (“late reduction at C-6”) but formed through partially separate pathways.

## 1. Introduction

**Figure 1:**
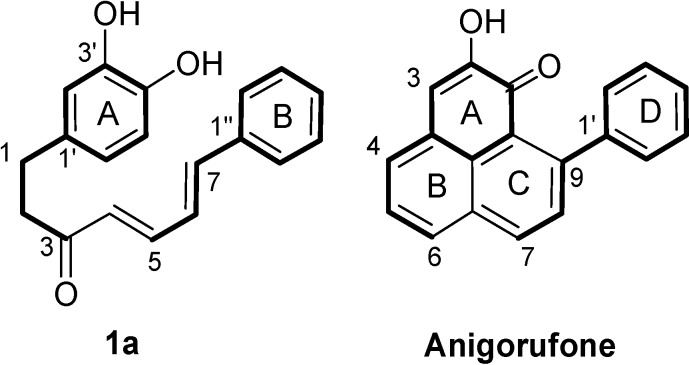
Structures of (4*E*,6*E*)-1-(3,4-dihydroxyphenyl)-7-phenyl-hepta-4,6-dien-3-one (**1a**) and anigorufone.

**Figure 2:**
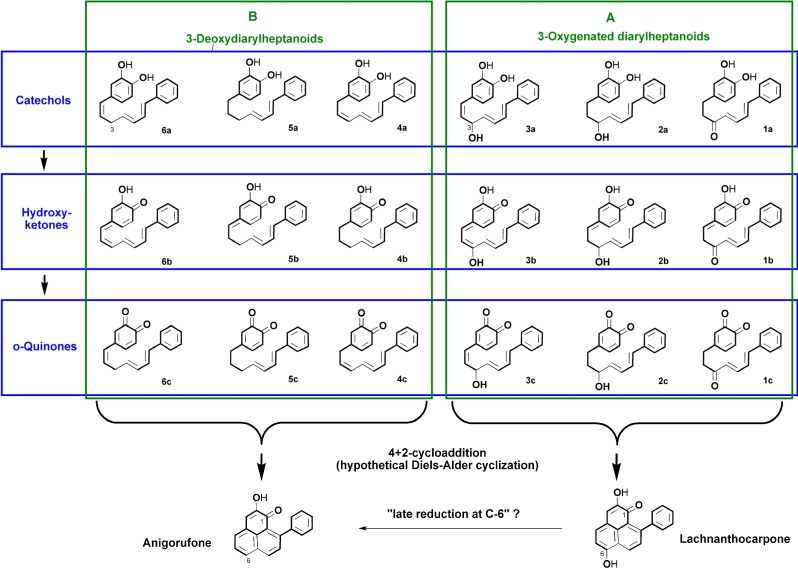
Diarylheptanoids potentially involved in phenylphenalenone biosynthesis.

Phenylphenalenones are a group of polycyclic plant natural products mainly occurring in the Haemodoraceae [1,2,3,4] and the Musaceae [5,6,7,8]. There is ample evidence that the biosynthesis of phenylphenalenones, first discussed by Thomas in 1961 [9], involves a diarylheptanoid, which undergoes cyclization to form the substituted tricyclic phenylphenalenone. This hypothesis was substantiated experimentally by the conversion of 1-(3,4-dihydroxyphenyl)-7-phenylhepta-4,6-dien-3-one (**1a**, Figure 1) to lachnanthocarpone in a one-pot chemical reaction [10] at ambient temperature. The authors proposed the electron-withdrawing *ortho*-quinone **1c** (Figure 2) as an intermediate, in which the 5'-en-3',4'-dione moiety functions as a dienophile. In addition to the *ortho*-quinone ring, the structure of the linear chain is of special interest for determining which diarylheptanoids can undergo (4+2)-cycloaddition. The 4,6-diene-3-one unit and a saturated segment in the C_7_-chain are characteristic features, which are hypothetically required to allow the Diels-Alder (DA) reaction to take place. If candidate diarylheptanoids would be available, then whether they could be experimentally converted to phenylphenalenones could be checked. The conversion could proceed either by chemical reaction *in vitro* [10], starting from compound **1a**, or *in vivo* after administration to plant material [11]. [2-^13^C]1-(3,4-Dihydroxyphenyl)-7-phenylhepta-4,6-dien-3-one (**1a**), when administered to cultured roots of *Anigozanthos preissii*, was smoothly converted to [8-^13^C]anigorufone [11].

According to the formation of lachnanthocarpone (*in vitro* chemical reaction, [10]) and anigorufone (*in vivo* biosynthesis, [11]) from **1a**, this compound seems to be a biosynthetic precursor of candidate substrates or a candidate structure itself for the DA reaction. However, other diarylheptanoids with varied substituents in ring A (catechol, hydroxyketone, *ortho*-quinone) or in the C_7_-chain (e.g., 1,3-diene-, 1,3,5-triene-, 5-oxo-, 5-hydroxy-) might be able to undergo the DA reaction either under chemical conditions or in the plant. The synthesis, preferentially in an isotopically labeled form, of a complete set of diarylheptanoids possessing combinations of different structural features, followed by chemical and biosynthetic experiments for conversion into phenylphenalenones, would be very laborious. Therefore, a computational approach has been applied to calculate the conformation of plausible candidates and the highest occupied molecular orbital (HOMO)/lowest occupied molecular orbital (LUMO) energy of the diene and the dienophile of a series of diarylheptanoid structures in order to estimate their suitability for cyclization.

## 2. Results and Discussion

### 2.1. Diarylheptanoid Candidate Structures for Diels–Alder Cyclization

Oxygen functionalities play an important role in the formation of phenylphenalenones from their open-chain diarylheptanoid precursors [12]. The oxidation stage of the functional groups affects the electron density at the corresponding carbon atoms of the aromatic ring A and in the C_7_-chain, and seems to facilitate cyclization to phenylphenalenones. The oxidation pattern of ring A, *i.e.*, the hydroxyketone or dihydroxy moiety at C-1/C-2, is a common feature of anigorufone (Figure 1) and most other phenylphenalenones. During biosynthesis, this *ortho*-dioxygenation pattern is retained from ring A of the open-chain diarylheptanoid precursor [12]. The two oxygen substituents in the *ortho* position (C-3'/C-4') of linear diarylheptanoids seem to be required or at least beneficial for the intramolecular DA cyclization because they are withdrawing electrons from the dienophile double bond (*i.e.*, C-5'/C-6') (structural criterion I). Therefore, three series of diarylheptanoids with differently 3'/4'-substituted ring A, namely catechols, hydroxyketones, and *ortho*-quinones, have been employed for computation (Figure 2).

However, an *ortho*-oxygen-substituted aryl ring is not the only requirement for cyclization. Curcumin from *Curcuma longa*, the most well-known diarylheptanoid, for example, is not converted to phenylphenalenones despite the 3-methoxy-4-hydroxyphenyl structure of the aromatic rings (Schneider, unpublished data). Thus, structural features associated with the C_7_-chain are also important prerequisites for cyclization. First, the properties of the diene (position of conjugated double bonds at C-4 and C-6 within the C_7_-chain and their conformation) have to be considered (structural criterion II). The conformation of the diene double bonds was *a priori* assigned cisoid in all candidate structures. Transoid conformation is inappropriate for the DA reaction and therefore was not taken into account. Moreover, to undergo (4+2)-cycloaddition, the diene and the dienophile have to be arranged in a non-planar, *i.e.*, stacked, geometry, as this arrangement facilitates the *exo*-orientation of the two moieties within the transition state. Stacked geometry requires a flexible C_7_-chain, which results from the occurrence of sp^3^-carbon atoms [13]. Thus, the occurrence of a saturated segment in the C_7_-chain next to ring A was defined as a third prerequisite (structural criterion III) for the DA reaction of diarylheptanoids.

The presence (e.g., 3-oxygenated diarylheptanoids, panel A in Figure 2) or absence of oxygen (e.g., 3,5-dideoxydiarylheptanoids, panel B in Figure 2) in the C_7_-chain may also play a role in determining the suitability of a diarylheptanoid for the DA reaction. The reduction of a hydroxyl group at C-5 of diarylheptanoids (5-OH originates from a carboxyl group of a phenylpropanoid) is assumed to take place early in biosynthesis, because phenylphenalenones with oxygen at the corresponding position (C-7) have so far not been reported from natural sources. The oxygen at C-3 of diarylheptanoids (3-OH originates from the carboxyl group of the second phenylpropanoid unit) is retained at C-6 of some phenylphenalenones (e.g., lachnanthocarpone) but is lost in others (e.g., anigorufone).

The computations envisaged in this study are thought to answer the question, which out of the 18 diarylheptanoid structures shown in Figure 2 are able to be folded into a geometry that allows the diene and the dienophile to be located at the distance required to undergo the suprafacial (4+2)-cycloaddition and which therefore is the preferred candidate biosynthetic precursor of phenylphenalenones. A conclusive answer to this question would help determine whether all of the different phenylphenalenones in plants are formed from a common diarylheptanoid or if different diarylheptanoids could function as precursors of different structural types of phenylphenalenones. In this context the question arose: Are 1,2-dioxygenated and 1,2,6-trioxygenated phenylphenalenones, exemplified by anigorufone and lachnanthocarpone (Figure 2), respectively, formed through a linear pathway or a grid of pathway variants?

### 2.2. Molecular Geometry Calculation

As outlined above, the following structural criteria seem to be hypothetical prerequisites for the intramolecular DA reaction of diarylheptanoids and have been employed as minimal conditions for computations:

- Two oxygen substituents in ortho position (C-3'/C-4') of linear diarylheptanoids (as in all diarylheptanoids shown in Figure 2).
- Conjugated double bonds at C-4 and C-6 in the C_7_-chain in cisoid conformation (as in all diarylheptanoids shown in Figure 2).
- A saturated segment (sp^3^-carbon atoms) in the C_7_-chain next to ring A.

**Figure 3:**
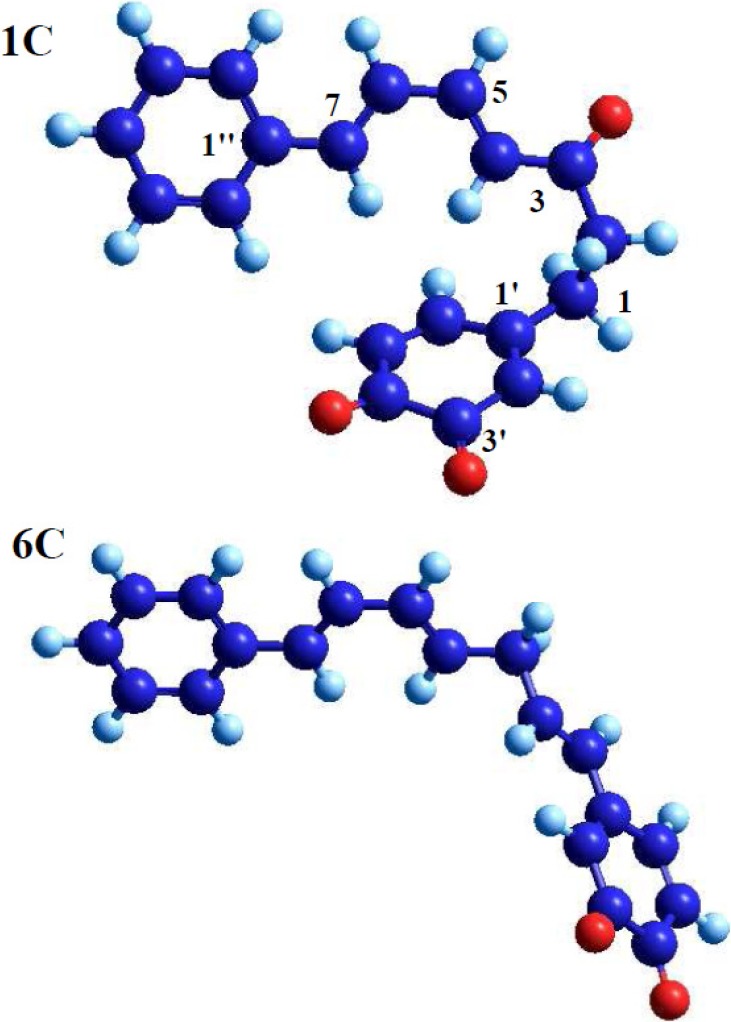
Diarylheptanoid **1c** and **6c** exemplify the two geometrical types of diarylheptanoids under study (the atom numbers in accordance with Figure 1 are shown for compound **1c**). Compound **1c** is bent into a stacked geometry with a short distance between the diene carbons C-4/C-7 and the dienophile carbon atoms C-5'/C-6' and therefore seems able to undergo the Diels-Alder cyclization. The stretched geometry of compound **6c** does not favor an intramolecular Diels-Alder reaction.

The compounds shown in Figure 2 were subjected to quantum chemical calculations, whether or not in each case they fulfill all of the above-mentioned structural criteria. Based on the calculated geometry of the optimized molecules (basis 6-31G*), the candidate structures can be categorized into two groups according to the “geometrical” criterion III, *i.e.*, having a saturated segment of sp^3^-carbon atoms next to ring A in the C_7_-chain. Compounds **1a** and **1c** (sp^3^-carbon atoms C-1 and C-2) and compounds **2a**, **5a**, **4b**, **2c** and **5c** (three sp^3^-carbon atoms C-1, C-2 and C-3) fulfill this particular criterion and are marked with “+” in Table 1. These compounds are bent into a stacked geometry (see **1c** in Figure 3 as an example). In contrast, the optimized structures of compounds **3c**, **4c** and **6c**, marked with “–” in Table 1 have only one sp^3^-carbon atoms in the chain at C-1 or C-3 and therefore show a stretched geometry or are only slightly bent (see **6c** in Figure 3 as an example). As expected, none of the other eight candidates (**3a**, **4a**, **6a**, **1b**, **2b**, **3b**, **5b**, **6b**) for which structure criterion III is not fulfilled is bent into a stacked geometry (Table 1). Hence, the presence of at least two sp^3^-carbon atoms in the C_7_-chain next to ring A seems to be essential for the intramolecular DA reaction.

**Table 1:** Geometrical and charge characteristics of the investigated compounds.

Compound	Geometrical Criterion ^a^	Distance Between Atoms [Å]		Mulliken’s Charges				Difference between Atoms’ Charges	
		(6'-4)	(5'-7)	6'	5'	4	7	(6'-4)	(5'-7)
**1a**	+	3.39	4.01	−0.11	−0.12	−0.29	−0.07	0.16	0.06
**2a**	+	3.48	4.07	−0.11	−0.12	−0.19	−0.11	0.08	0.02
**3a**	−	− ^b^	− ^b^	−0.08	−0.12	−0.17	−0.10	0.09	0.02
**4a**	−	− ^b^	− ^b^	−0.09	−0.12	−0.11	−0.01	0.02	0.02
**5a**	+	3.46	4.11	−0.11	−0.12	−0.16	−0.11	0.05	0.01
**6a**	−	− ^b^	− ^b^	−0.09	−0.12	−0.14	−0.10	0.05	0.02
**1b**	−	− ^b^	− ^b^	−0.03	−0.26	−0.21	−0.05	0.18	0.21
**2b**	−	− ^b^	− ^b^	−0.03	−0.21	−0.18	−0.09	0.15	0.12
**3b**	−	− ^b^	− ^b^	−0.01	−0.22	−0.15	−0.06	0.15	0.16
**4b**	+	3.47	4.10	−0.09	−0.19	−0.17	−0.10	0.08	0.09
**5b**	−	− ^b^	− ^b^	−0.04	−0.21	−0.15	−0.10	0.11	0.11
**6b**	−	− ^b^	− ^b^	−0.02	−0.21	−0.14	−0.08	0.13	0.13
**1c**	+	3.38	3.93	−0.05	−0.20	−0.27	−0.06	0.22	0.14
**2c**	+	3.50	4.02	−0.05	−0.19	−0.19	−0.10	0.14	0.09
**3c**	−	− ^b^	− ^b^	−0.05	−0.19	−0.18	−0.09	0.13	0.10
**4c**	−	− ^b^	− ^b^	−0.05	−0.19	−0.12	−0.09	0.08	0.10
**5c**	+	3.47	4.04	−0.05	−0.19	−0.17	−0.10	0.11	0.10
**6c**	−	− ^b^	− ^b^	−0.05	−0.19	−0.15	−0.09	0.11	0.10

^a^ based on visual inspection of the optimized geometry; ^b^ not quoted as geometry condition was not fulfilled (the distances were more than 7 Å).

For structures showing bent geometry, the distances between the carbon atoms C-5'–C-7 and C-6'–C-4, which are participating in the cyclization, have been calculated (Table 1). This parameter varied between 3.93 Å and 4.11 Å for C-5' and C-7 and between 3.38 Å and 3.50 Å for C-6' and C-4. In both cases the values were the smallest for **1c**. It can be concluded that, although these distances are only slightly larger for the other compounds, compound **1c** is the most privileged candidate for the DA reaction.

Mulliken’s charges of the interacting atoms C-5', C-6', C-4, and C-7 are another important parameter to be considered. Table 1 contains the absolute values of Mulliken’s charges on the investigated atoms. According to the theory [14,15], the dienophile carbons should have low electron density. This is consistent especially for C-5' of most compounds and more or less also for C-6', although compound **1c** is not among the best examples in this case. In contrast to the dienophile, the diene component should be electron-rich, which is not the case for the considered compounds. However, the rule of electron depletion for the dienophile and electron excess for the diene can be reversed in so-called inverse electron-demand DA reactions. Table 1 also presents charge differences for C-6'–C-4 and C-5'–C-7. Clearly, the bigger this difference is, the stronger the interaction of the two atoms and the more favored the DA reaction. Compound **1c** has the largest charge differences between both positions C-6'–C-4 (0.22) and the third largest difference for the other pair of atoms C-5'–C-7 (0.135). The smallest charge differences were found for compounds from group **a** (catechols), namely in compound **5a** for atoms C-5'–C-7 (0.01) and in compound **4a** for C-6'–C-4 (0.02).

### 2.3. Orbital Calculation

**Figure 4:**
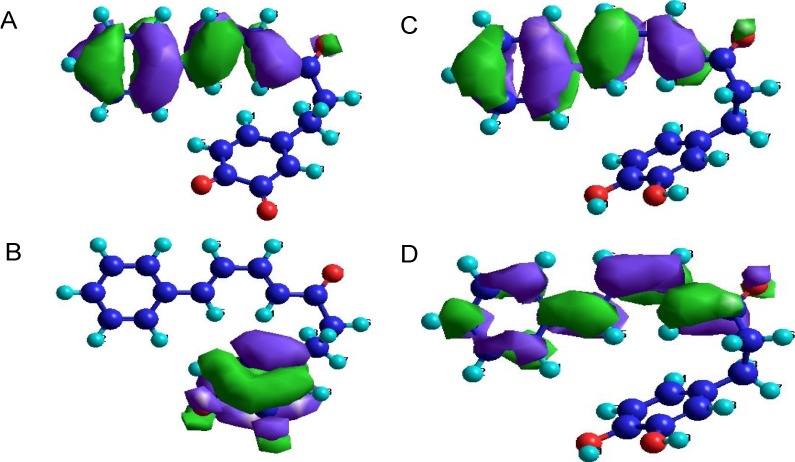
(**A**) HOMO and (**B**) LUMO of compound **1c** and (**C**) HOMO and (**D**) LUMO of compound **1a**.

Another aspect to be considered for the feasibility of the DA reaction is the interaction of HOMO-LUMO and the characteristics of these orbitals. Table 2 shows energy values of both HOMO and LUMO and the difference between them for all structures under consideration. There is a clear tendency: the biggest differences were found for catechols (Figure 1, group **a**), then three hydroxyketones (**1b**, **2b** and **5b**), followed by compounds from group **c** (*ortho*-quinones). Interestingly, compounds **3**, **4**, and **6** from groups **b** and **c** have the smallest HOMO-LUMO energy differences. Figure 4 displays HOMO and LUMO of compounds **1a** and **1c** and illustrates their possible interactions. The DA reaction seems to occur if p-orbitals of the dienophile atoms C-6' and C-5' give the largest impact in HOMO and p-orbitals of the diene atoms (C-4 and C-7) in LUMO, or vice versa. This is the case for compounds **1c**-**6c** as well as for some compounds from group **b** (**2b**, **4b** and **6b**) (Table 2). The other studied compounds do not fulfill this condition. For compound **1a**, for example, p-electron density concentrates on dien chain (C-4 to C-7) and ring B (Figure 4).

**Table 2:** Characteristics of LUMO and HOMO energies for selected compounds.

Compound	Orbital Criterion ^a^	Energy of Orbitals [eV]			Compound	Orbital Criterion ^a^	Energy of Orbitals [eV]		
		LUMO	HOMO	LUMO-HOMO Difference			LUMO	HOMO	LUMO-HOMO Difference
**1a**	−	−0.87	−8.94	8.07	**4b**	+	−0.42	−5.17	4.75
**2a**	−	−0.34	−8.55	8.21	**5b**	−	−1.21	−8.76	7.55
**3a**	−	−0.49	−8.66	8.17	**6b**	+	−1.73	−8.31	6.58
**4a**	−	−0.70	−8.45	7.75	**1c**	+	−1.80	−9.15	7.35
**5a**	−	−0.34	−8.53	8.19	**2c**	+	−1.69	−8.76	7.07
**6a**	−	−0.52	−8.58	8.06	**3c**	+	−1.77	−8.81	7.04
**1b**	−	−1.37	−9.10	7.73	**4c**	+	−1.59	−8.66	7.07
**2b**	+	−1.29	−8.74	7.45	**5c**	+	−1.63	−8.74	7.11
**3b**	−	−1.74	−8.16	6.42	**6c**	+	−1.67	−8.66	6.99

^a^ based on visual inspection of LUMO and HOMO orbitals.

This finding was numerically substantiated by calculating contributions of all p-orbitals in HOMO and LUMO for C-5', C-6', C-4, and C-7 (Table 3). For example, for compound **1c** the interactions C-7 (p_x_)–C-6' (p_y_) and C-5' (p_x_)–C-4 (p_y_) are possible. On the other hand, for compound **2a**, for example, p-orbitals of C-6' and C-5' do not play significant roles in HOMO and LUMO. From this point of view, compounds **1c**–**5c**, as well as **2b**, **4b** and **6b** are suitable candidates for the intramolecular DA reaction. Although the “orbital” criterion is also fulfilled for compounds **3b** and **6c**, the impact of p-orbitals of C-5' in LUMO (**6c**) and of C-4 in LUMO (**3b**) is very low and, therefore, the desired HOMO-LUMO interaction is unlikely.

This shows that the calculation of HOMO and LUMO of the positions involved in the intramolecular DA reaction alone does not allow the viability of the diarylheptanoids under study to be predicted. Instead, it is necessary to consider the combination of the geometrical and orbital parameters. From the data in Table 1, Table 2 and Table 3 it can be concluded that for some compounds (**1a**, **2a**, **5a**, **4b**, **1c**, **2c**, **5c**), the “geometrical” criterion III shows that the reaction is possible, whereas for other compounds (**2b**, **6b**, **3c**, **4c**, **6c**) orbital characteristics point to reaction possibility. Taking into account both factors, compounds **1c**, **2c**, **5c**, and **4b** seem to be suitable for the intramolecular DA reaction to form phenylphenalenones.

## 3. Experimental

Quantum-Chemical Calculations: We used GAMESS (US) v.7.0 [16,17,18] and HyperChem Professional v.8.0 (Hypercube, Gainesville, FL, USA) software packages for quantum chemical calculations. First, we applied semi-empirical PM3 (Parametrised Model 3) method with full geometry optimization to obtain the rough geometry of molecules. The main approaches of the PM3 method include adiabatic, one-electron, MO LCAO (molecular orbital as a linear combination of atomic orbitals) and INDO (Intermediate Neglect of Differential Overlap) approximations. For details regarding the calculations, see [19]. We were particularly careful to ensure that the diene double bonds had a cisoid conformation, as this would be favorable for the DA reaction (Figure 1). All structures were then optimized at the unrestricted Hartree-Fock (UHF) [20] level of theory using the 6-31G* [21] basis set and PM3 geometry as inputs. This level of theory is sufficient for calculating the selected parameters (Mulliken’s charges, distances between atoms, energy of HOMO and LUMO orbitals) for a series of similar compounds.

**Table 3:** Contribution of the most important atomic orbitals of carbon atoms in LUMO and HOMO (for atom numbers, see Figure 1).

Compound	Atom number	Atomic orbital	Type of molecular orbital	Contribution	Compound	Atom number	Atomic orbital	Type of molecular orbital	Contribution
**1a**	7	Px	LUMO	0.23	**4b**	7	Px	LUMO	0.24
	4	Px	LUMO	0.19		4	Px	LUMO	0.24
	5'	Px	HOMO	0.00		5'	Px	HOMO	0.03
	6'	Py	HOMO	0.00		6'	Pz	HOMO	0.37
**2a**	7	Py	HOMO	0.20	**5b**	7	Pz	LUMO	0.03
	4	Px	HOMO	0.24		4	Pz	HOMO	0.32
	5'	Pz	LUMO	0.00		5'	Px	LUMO	0.31
	6'	Pz	HOMO	0.01		6'	Px	HOMO	0.03
3a	7	Px	HOMO	0.21	**6b**	7	Pz	HOMO	0.21
	4	Py	HOMO	0.27		4	Pz	LUMO	0.12
	5'	Pz	LUMO	0.03		5'	Pz	LUMO	0.20
	6'	Px	HOMO	0.07		6'	Pz	HOMO	0.16
4a	7	Py	HOMO	0.33	**1c**	7	Px	HOMO	0.17
	4	- ^a^	- ^a^	- ^a^		4	Py	HOMO	0.08
	5'	Px	HOMO	0.00		5'	Px	LUMO	0.045
	6'	Pz	LUMO	0.01		6'	Py	LUMO	0.11
**5a**	7	Pz	LUMO	0.24	**2c**	7	Py	HOMO	0.20
	4	Px	HOMO	0.24		4	Px	HOMO	0.23
	5'	S	LUMO	0.00		5'	Px	LUMO	0.09
	6'	Pz	HOMO	0.00		6'	Pz	LUMO	0.36
**6a**	7	Pz	LUMO	0.27	**3c**	7	Py	HOMO	0.28
	4	Py	LUMO	0.26		4	Px	HOMO	0.21
	5'	Px	LUMO	0.02		5'	Pz	LUMO	0.13
	6'	Px	HOMO	0.26		6'	Px	LUMO	0.25
**1b**	7	Py	HOMO	0.08	**4c**	7	Pz	HOMO	0.33
	4	Pz	HOMO	0.28		4	Pz	LUMO	0.02
	5'	Px	HOMO	0.32		5'	Py	HOMO	0.00
	6'	Px	LUMO	0.12		6'	Py	LUMO	0.24
**2b**	7	Px	HOMO	0.25	**5c**	7	Py	HOMO	0.22
	4	Py	HOMO	0.19		4	Px	HOMO	0.24
	5'	Pz	LUMO	0.11		5'	Px	LUMO	0.11
	6'	Px	LUMO	0.33		6'	Pz	LUMO	0.35
**3b**	7	Px	HOMO	0.09	**6c**	7	Py	HOMO	0.27
	4	Py	LUMO	0.03		4	Px	HOMO	0.22
	5'	Pz	LUMO	0.08		5'	Px	LUMO	0.27
	6'	Pz	HOMO	0.21		6'	Px	LUMO	0.01

^a^ no significant contribution of p-orbitals in HOMO and LUMO.

## 4. Conclusions

This computational study confirmed that for intramolecular Diels-Alder cyclization of diarylheptanoids to produce phenylphenalenones, both the dienophile located in ring A of the diarylheptanoid and the C_7_-chain, comprising the diene and sp^3^-carbon atoms, must possess optimal electronic and conformational characteristics: an *ortho*-quinone- or hydoxyketone-bearing ring A containing the dienophile moiety and a heptadiene chain with conjugated cisoid double bonds at C-4/C-6. According to the molecular geometry and orbital calculations, compounds **1c**, **2c**, **5c**, and **4b** are the best substrates for DA cyclization of diarylheptanoids to produce phenylphenalenones. The type of oxygen functionality at C-3 of the C_7_-chain seems not to be crucial for the DA reaction since both compounds **1c** and **2c** are good candidates. This result is in agreement with previous chemical and biosynthetic studies [10,11]. Interestingly, the absence of oxygen in the C_7_-chain of **4b** and **5c** does not prevent these diarylheptanoids from undergoing the intramolecular DA reaction. Hence, the possible DA reaction of both 3-oxygenated compounds **1c** and **2c** and 3,5-dideoxy compounds **4b** and **5c** suggests that 6-oxygenated phenylphenalenones, such as lachnanthocarpone and 6-deoxy phenylphenalenones, such as anigorufone, are not connected via a precursor-product relationship (“late reduction at C-6”) but formed through early reduction at C-6 via partially separate pathways. This hypothesis remains to be confirmed experimentally by biosynthetic labeling or enzyme studies.
